# Complete mitochondrial genomes for *Cottus asper*, *Cottus perifretum*, and *Cottus rhenanus* (Perciformes, Cottidae)

**DOI:** 10.1080/23802359.2017.1375870

**Published:** 2017-09-18

**Authors:** Kayla Fast, Andres Aguilar, Arne W. Nolte, Michael W. Sandel

**Affiliations:** a Department of Biological and Environmental Sciences, The University of West Alabama, Livingston, AL, USA;; b Department of Biological Sciences, California State University Los Angeles, Los Angeles, CA, USA;; c Department of Ecological Genomics, University of Oldenburg, Oldenburg, Germany

**Keywords:** Sculpin, *Cottus asper*, *Cottus perifretum*, *Cottus rhenanus*, mitochondrial genome

## Abstract

Freshwater sculpins represent a diverse but poorly-understood constituent of the Holarctic ichthyofauna. Sculpins are considered sensitive to pollution and habitat change, serving as aquatic bioindicators in ecotoxicology. Many species are protected by conservation agencies, due to anthropogenic activity within restricted geographic distributions. Here, we provide the first complete mitochondrial DNA sequences for three freshwater sculpins (*Cottus asper*, *C. perifretum*, *C. rhenanus*). These data are used to infer an updated mtDNA phylogeny for the genus *Cottus*, which supports results of previous research. These data are likely to be useful for future studies in biogeography, conservation, and functional genomics.

The genus *Cottus* contains at least 263 species of sculpins distributed in Holarctic freshwaters and, to a lesser extent, coastal marine environments (Boschung and Mayden 2004). The Prickly Sculpin (*Cottus asper*) inhabits Pacific versant watersheds of North America from Ventura River, California, northward to the Kenai Peninsula of Alaska (Krejsa 1965). The distribution of *C. perifretum* includes the Rhine River drainage in Germany, the Netherlands, and France; rivers in Great Britain; drainages from the Garonne to Scheldt and Meuse in France and Belgium; and the Mosel and Sieg Rivers in Germany (Freyhof et al. 2005; The IUCN Red List of Threatened Species 2016). *Cottus rhenanus* inhabits the Meuse River and the Rhine River drainage north to about Mannheim (Freyhof et al. 2005).

The complete mitochondrial genomes of the three freshwater sculpins were sequenced: *Cottus asper*, *C*. *perifretum*, and *C*. *rhenanus* (GenBank accession numbers MF326939, MF326940, and MF326941, respectively). *C. asper* was collected in Redwood Creek (GIS: 41°17′N −124°02′W; Humboldt County, CA), *C*. *perifretum* in Stream Witte Nete (GIS: 53°14′N 5°05′E; Flanders, Belgium), and *C*. *rhenanus* in Stream Broel (GIS: 50°50′N 7°22′E; North Rhine Westphalia, Germany). The *Cottus asper* voucher specimen is stored at California State University Los Angeles. Voucher specimens for *C. perifretum* and *C. rhenanus* were not retained, but voucher DNA is archived by A.N. at the University of Oldenburg. Mitochondrial genome sizes for *C. asper*, *C*. *perifretum*, and *C*. *rhenanus* were 16,511 bp, 16,523 bp, and 16,522 bp, respectively. Sequence data were generated using illumina HiSeq at Global Biologics (*C. asper*) and the Cologne Center for Genomics facility of the University of Cologne, Germany (*C. perifretum* and *C. rhenanus*).

Paired-end reads were assembled using MitoBim 1.7 (Hahn et al. 2013) using the –quick option and default settings, on the Cheaha cluster computer at the University of Alabama at Birmingham. The mitochondrial genome sequence of *Cottus poecilopus* (GenBank accession EU332750) was used as a reference sequence. Assembled mitochondrial genomes were manually validated and aligned with previously published Cottidae mitogenomes using Bioedit 7.0.0 (Hall 2005). MEGA version 6 (Tamura et al. 2013) was used to identify the optimal nucleotide substitution model (GTR + G+I; Nei and Kumar 2000) and conduct a phylogenetic analysis under the maximum likelihood optimality criterion (Figure 1). Minimum evolution and neighbour-joining trees resulted in the same tree topology as the maximum likelihood tree. With the exception of the taxa added in this paper, phylogenetic analyses revealed the same tree topologies (Figure 1) reported in recent studies of *Cottus* mitochondrial genomes (Balakirev et al. 2016; Han et al. 2016; Swanburg et al. 2016). Despite limited taxonomic coverage, this new *Cottus* topology suggests multiple independent invasions of Asia, Europe, and North America (Figure 1). Additional taxonomic sampling will aid in resolving the biogeography of this enigmatic group of freshwater fishes. Finally, novel SNPs identified from these three mitochondrial genomes may prove useful for rapid haplotyping in populations where genetic introgression is cause for concern by conservationists (Nolte et al. 2006; Baumsteiger and Aguilar 2014).

**Figure 1.**
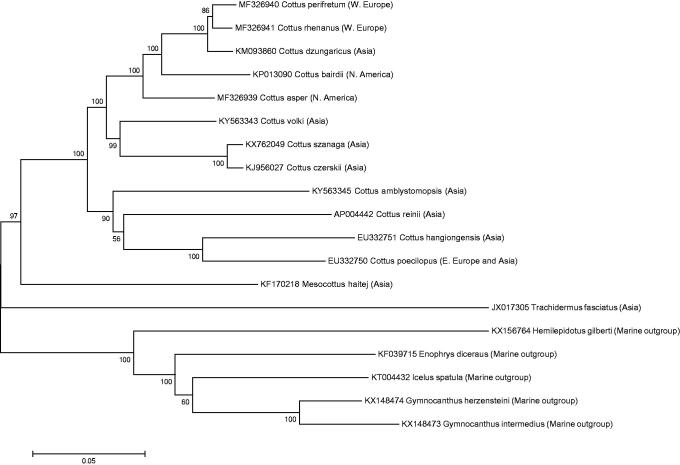
Interspecific phylogeny inferred under the Maximum Likelihood (GTR + G+I) optimality criterion; log likelihood = −91771.0521 (Nei and Kumar 2000). Support values represent the proportion of 500 bootstrap replicates in which the associated taxa clustered together. A discrete Gamma distribution was used to model evolutionary rate differences among 5 site categories (+G, parameter =0.9798), where the proportion of invariant sites (+I) equals 55.5%. Gaps and missing data were eliminated from a final alignment of 16284 base pairs. Evolutionary analyses were conducted in MEGA6 (Tamura et al. 2013).
